# Remarks on Cancer, and on Mr. Young's New Mode of Treating That Disease
*Mr. Young's work will be noticed in this or in our next Number. For that gentleman's former remarks on Cancer, see our Journal, vol. xiv. p. 465, and xv. p. 393.—Edit.


**Published:** 1815-11

**Authors:** Thomas Denman


*For the London Medical and Physical Journal.*

### *Remarks on Cancer, and on Mr. Young's* new Mode of Treating that Disease;* by Thomas Denman, M.D.

HAVING, for many years past, paid great attention to the subject of *Cancer*, and supposed cancerous diseases, it gave me much satisfaction to read Mr. Young's book, in which he has proposed a new method of treatment, supported by the history of many cases in which it had been practised with very great advantage. Since the publication of his book, I have had opportunities of seeing several under his own particular care, and have been informed of others, on which his method was tried, under the care and direction of different gentlemen; together with the effects which almost invariably follow his mode of treatment. I also beg leave to premise a few general observations on the subject.

The disease called *Cancer* has been for time immemorial distinguished by that appellation, and for almost the same extent of time has been considered as a living substance, so far as to be imagined to possess a living principle, independant of the life of the person afflicted with it; nor is the period known when the disease was first asserted to be incurable, but that opinion remains generally, with very few dissenting voices, to the present day.

---

* Mr. Young's work will be noticed in this or in our next Number. For that gentleman's former remarks on Cancer, see our Journal, vol. xiv. p. 465, and xv. p. 393.—Edit.

There

There have been two opinions concerning this disease, one, that it was local,—the other, that it was constitutional; and, according to the opinions entertained, have been the different modes of practice, the events of which have not induced an alteration of opinion.

Though the incurable nature of this disease has been generally allowed, the industry of the professors of the different branches of medicine has not slackened, for they have, with the most commendable motives, endeavoured, by all the powers of medicine, and by all the means they could devise, to overcome the difficulties with which they were struggling.

The chemists have reasonably made their attempts by exploring the component principles of parts affected with this disease, and of the discharges thence derived, by bringing them to the test of fire, in every way in which they could be tried.

The anatomists have, with equal industry and sagacity, exerted themselves to discover the structure of parts affected with cancer, at every period of the disease; and in this they have certainly succeeded by a most exact description. They have, moreover, discovered certain filaments which make constituent parts of cancer, though some have doubted whether these were peculiar to that disease.

Nor have physicians, who seem to be of the opinion that the disease is constitutional, been deficient in their endeavours to discover a remedy for it, having exhibited almost every medicine which promised either an effectual cure for the disease, or to afford relief.

Surgeons who have been most frequently consulted in these cases, especially at their commencement, seem to have placed their chief confidence in the extirpation of the diseased part, by the knife, or by caustic applications. But, in these operations, which were most severe and painful, there had been so many failures,—that is, the disease had so often returned, that surgeons who were circumspect in their conduct, and had witnessed such failures, used so much caution in giving their consent to the performance of operations, as almost to amount to a prohibition. But every surgeon who has performed such operations, must have considered the disease as local; otherwise he deprived himself of the only justifiable reason for performing them.

With regard to the kind of life which cancer was thought to possess, some have supposed it to be similar to that of the insect from which it derived its name; some that it resembled that of the hydatid;* while others supposed it to be like

* On the Hydatid Life of Cancer. See Mr. Young's Correspondence with one of our Editors, vol. xv. p. 393, and vol. xvi. p. 97.

that of a mushroom, which is thought to partake of the properties of the animal and vegetable tribe. With regard to the last conclusion, there certainly is, from the commencement to the conclusion of cancer, a great disposition to generate fungus. Whatever was the opinion while the disease remained in possession of its original powers, it was, and is still, supposed to be incurable. When, therefore, operations were not performed, and were not approved, attempts were made to cure the disease by different outward applications, the greatest part of which were composed of very active or poisonous ingredients, which were supposed to have the power of destroying it, but which also failed.

Here, then, the matter stood, when Mr. Young brought forward his method of treatment, for *his* I must consider it, even if it had been used before, it was so little known as to have become obsolete or forgotten. By his method, some very unexpected occurrences are produced. Whatever was the degree of pain which the patient before suffered, that is speedily appeased; and, however offensive the discharge may have been previously, this becomes simple and void of smell, excepting what is usual in common sores. Now, if, by any other means, equally innoxious, such effects could be produced, I might hesitate which to prefer; but, as we are, I believe, ignorant of any such means, certainly these may be considered as great advantages. But the matter does not rest here, for there is an almost instant stop to the ravages of the disease, whether occult or open, according to the ancient expression; the discharges are gradually lessened, and from the appearance of being of a most acrimonious kind, become bland and salutary. Besides, there is evidently a gradual decrease of the tumefaction and induration; and the whole not only assumes the appearance of amendment, but a promise of perfect recovery, as is proved in several cases which I have seen.

I have heard two reasons advanced in opposition to Mr. Young's method;—first, that other diseases, equally dangerous, may be produced by it; secondly, that, though, by his method, cancer of the breast may be cured, it cannot be applied to many other parts affected with that disease. These seem to me mere presumptions, not arguments supported by any experience we yet have of his method. It would be equally or more fair to consider his method as standing on a principle which may lead to the cure of other diseases; for instance, of the *fungus hæmatodes*, so acutely discovered, and so correctly described, by Mr. Hey, of Leeds; and, perhaps, to many others.

Should this method of Mr. Young's be discountenanced and neglected without a fair and unprejudiced trial, I fear much injury may be done to society; to the professors of medicine in general, by abasing their most humane character; and real injustice to a very intelligent and meritorious individual.

*Mount-street; October* 9th, 1815.

